# Report of the Standing Sanatory Committee of the Board of Health of the City of New York, on the Subject of Asiatic Cholera, at Present Prevailing at the Quarantine Establishment of New York, at Staten Island

**Published:** 1849-02

**Authors:** 


Art. X.—*Report of the Standing Sanatory Committee of the Board of Health of the City of New York,* on the subject of Asiatic Cholera, at present prevailing at the Quarantine Establishment of New York, at Staten Island. New York: McSpedon & Baker. 1848. Pamphlet.

Of all the subjects at present occupying the public mind, that which excites the deepest and most pervading interest is unquestionably cholera. A very general and painful apprehension is felt that the epidemic is impending the country and will break out on the return of warm weather. Under these circumstances it may be presumed that the attention of physicians is everywhere anxiously turned to the disease, and that they are industriously preparing themselves to meet it. At two points in our country it has made its appearance, and at one has prevailed extensively in a malignant form. Up to the present moment we are without any professional account of the epidemic in that city, but the newspapers contain the reports of the Board of Health of the City of New Orleans, from which we are able to gather some interesting particulars. The Report before us affords a history of the disease as it prevailed at New York.

From this Report it appears that the packet ship New York arrived at Quarantine, on the 2d of December, with a number of sick persons, having lost seven during the last week of the voyage, with a disease now admitted to be Asiatic cholera. The ship left Havre on the 9th of November with 331 steerage passengers, 21 cabin, and 33 crew; a total of 385. All continued well until the 25th, when one of the steerage passengers, a German, aged 29 years, in robust health, was attacked with vomiting and purging, accompanied by cramps of the muscles of the upper and lower extremities. He died on the third day, although "the captain prescribed judiciously for the symptoms." The day following an old man, in feeble health, was attacked with vomiting and purging, with coldness of the whole body, and violent spasms, of which he died on the second day after the attack. In the two succeeding days two cases occurred, one in a little girl, 5 years old, who died in two hours, and the other in a boy of the same age, who died in four and a half hours after their first attack, both previously in good health. The next day, a man aged 40 years was attacked at 8 o'clock, A.M., and died at 3 P.M., of the same day; and on the day after, two children sickened and died after six and eight hours illness. The ship came to anchor at Quarantine on the night of the 1st of December, and from that time until noon of the 3d, when the passengers were landed, twelve new cases occurred.

Since the arrival of the ship, the Report continues, 18 cases have occurred, making, with the 12 taken from the ship, 30 cases in all, of which 20 have terminated fatally. The whole number from the first case at sea, has been 37, of which all but 10 have had a fatal termination. No recovery took place until the passengers were taken ashore and the sick placed under the care of physicians.

As to the symptoms ushering in the disease, Dr. Whiting, the Health Officer at Quarantine, and author of this Report, says diarrhea preceded the attack in only about one-third of the cases. Vomiting and purging were present, the matters discharged being "well described as *rice-water* evacuations." Great uneasiness and pain, particularly at the epigastrium, attended the vomiting, which sometimes existed without purging, and *vice versa.* In several cases although neither vomiting nor purging occurred, the stomach and bowels were found, after death, filled with the peculiar fluid. The tongue and breath are described as having been "icy cold;" lumbrici were discharged in a large majority of cases; the skin, countenance, voice, and extremities, exhibited the characters so well known in this disease. Cramps affected all the subjects, and generally were a very painful symptom. The number or apparent violence of the symptoms formed no criterion for the prognosis, fatal results following in a number of cases, in a few hours, when the evacuations were slight, and spasms and other violent symptoms were absent.

"In this disease there has been but one stage—that of collapse—although every pains have been taken to detect the first deviations from health, directions given to all to communicate them at once, and persons employed to inspect them constantly, and a physician to pass among them at all hours of the night and day. The first intimations are the extreme symptoms, defying the most prompt and decided remedies."

At the time of the arrival of the packet referred to, nothing like cholera existed at Staten Island, or in the city of New York. A number of cases afterwards appeared among the convalescent patients at Staten Island, some of whom were in communication with the sick removed from the ship. A man just recovering from a fractured patella, assisted in their removal, was attacked with violent symptoms of cholera, and died the same day. Others were seized in adjoining apartments, without any communication with the cholera patients. Except these cases among the inmates of the hospital, all the persons attacked from first to last were Germans, who had been living in Havre and its environs, where no cholera was prevailing at the time of their departure. But, says a writer in the New York Courier and Enquirer, many of these emigrants were from Hamburgh and other parts of Germany where the disease did prevail. "One or more of these poor people," this writer supposes, "brought with them the bedding or clothes in which one of their friends had died of cholera." The state of the weather in which the disease broke out at sea may be noted.

"At the time the first cases occurred, November 25th, the ship was in N. lat. 42°, long. 61°, about 140 miles S. S. W. from Sable Island. On the 23d and 24th, the two days preceding the appearence of the cholera, the wind was N. N. W. On the 25th it changed to the southward, with squalls and rain. In the morning the barometer was at 30 inches, and fell during the day to 29½ inches; thermometer 60° Fahrenheit. Sunday and Monday, 26th and 27th, wind westerly, and fresh; Tuesday, 28th, moderate from N. W.; barometer 30, thermometer 42°."

The first part of the voyage being mild, no extra clothing was required, but on reaching the cold, raw atmosphere of the Grand Banks, continues the writer above alluded to, it was necessary to put on a warmer dress, "when open comes the big chest and out comes the infected clothing into a close atmosphere reeking with the foul air and filth of 350 closely-stowed emigrants. This is the plain reason why the vessel was at sea seventeen days before the disease made its appearance, and this explains the eccentric movements of the disease on land."

Dr. Whiting does not attempt to account for the origin of cholera in the ship, and is not satisfied with his success in its treatment, at which we do not wonder when, in so many cases, the malady "presented its first and final stages simultaneously." His general plan, which was successful in some instances, was to envelop the patient in warm blankets, apply mustard extensively to the body and extremities, and aid its application by hot bricks, bags of sand, etc., and a stream of hot air conveyed under the bed clothes. Friction with hot tincture of capsicum was at the same time carried on under the bed clothes, in a way not to expose the body to the air. Mustard emetics were tried, but in only two cases did they seem to be beneficial. In eight cases, the large doses of calomel, capsicum, and camphor, of Dr. Cartwright, were carefully tried, aided by the external use of the hot tincture of capsicum, but the results were not satisfactory.

"Chloroform has been administered in a number of cases, carefully and repeatedly, and at first gave some hope that it would prove a successful remedy, but no other permanent good has resulted from its use but to relieve the spasms and cramps. For this purpose I have used it in all cases moderately, and if not a cure for all the symptoms, it is an invaluable remedy in subduing one of the most painful symptoms of the disease."

The *saline mixture* he found "worse than inefficient," and sugar of lead and opium, in large and small doses, were "soon abandoned as impotent."

"The treatment that has proved of most service has been calomel in scruple doses, combined with opium and camphor, followed at two or three hours intervals, by smaller doses of calomel, until reaction is indicated by some action of the liver. This plan, combined with the faithful application of external heat, &c., I am satisfied has proved of most advantage in the cases that have come under my notice. Every case in which the slightest bilious evacuation has been procured, has commenced to recover from that moment, and although of itself, unable to effect the reaction necessary for its own peculiar action, calomel will doubtless always prove the most potent auxiliary in the catalogue of remedies for cholera."

On the 19th of December Dr. Whiting further reported, that, since the 11th of the month, when his first report was made to the Board of Health, thirty-three new cases of cholera had occurred, and all but three among the German emigrants. Of the three, one was a Frenchman, from Paris, and two were inmates of the hospital, just recovering from typhus fever, who had no intercourse with the cholera cases. He adds:

"The whole number of cases, thus far at Quarantine, has been sixty-three. Of these twenty-nine have died. A large proportion have been children under fourteen years, twenty, or about one-third of the whole number having been of this class.

"Most of them passed through the first attack of the disease, and died from subsequent congestion or effusion in the brain.

"Of the thirty-three cases that have happened since my report of the 11th, I am glad to state that only nine have been fatal. And as there appears to be no difference in the severity of the symptoms at the outset of the disease, I cannot but attribute the diminished fatality to a more happy plan of treatment."

The results of the first thirty cases, with the post-mortem appearances, convinced Dr. Whiting "that the stimulating plan was not the treatment for *this* cholera," and accordingly he abandoned, first, the mustard, and then successively the capsicum, ammonia, brandy, wine whey, etc., and relied on calomel in moderate doses, with opium, Dover's powder, and camphor. Camphor he reregards as of questionable utility. He says, in conclusion—

"The treatment I have now adopted and adhere to, from its decided agency in controlling the symptoms and inducing early reaction, is the exhibition of moderate doses of calomel, with morphine, at short intervals. Five grains of calomel, with a quarter of a grain of sulph. morphia, is at first given to an adult; in half an hour, or one hour, a scruple dose of calomel is exhibited, and is usually retained; afterward a pill of Cal. grs. v. Sulph. Morph. gr. $\frac{1}{4}$, is given each hour, two hours or three hours, as the effect may indicate. This is observed in the subsidence of the pain and spasms, the diminished quantity and frequency of the evacuations, the return of warmth, and the restoration of the pulse.

"This treatment is continued until some indications of bilious action appear; the first is usually a change of color and consistence from the light, thin, rice water, to a greenish, and then brown or brownish yellow color. The evacuations from the stomach and bowels will frequently continue green, or of the color of sulphate of copper, for hours, but I have not known a single case to relapse where this effect had once been produced."

We have no doubt, from all that we have seen of cholera and read respecting the disease, that this is the best treatment. In mild cases it may be sufficient to restrain the diarrhea by stimulants and astringents, but we believe we are safe in saying that all American experience is to the effect, that the only method to be relied upon in the disease is one which induces action in the liver. When the passages become bilious we regard our patients as safe, and not before. Such was the conclusion to which, with remarkable unanimity, the minds of the physicians of our country came during the prevalence of the epidemic in 1832, and subsequently; and notwithstanding all that has been since said by English practitioners in favor of other plans of treatment, the mercurial is the one to which we should still resort. We are glad to find that we are sustained in this conviction by the experience of the Health Officer at Staten Island. Calomel, given with brandy, or in combination with morphia or Dover's powder, and aided by hot vapor, and hot mustard poultices, with chloroform to excite warmth and restrain the spasms, promises more, in our judgment, than any other method of cure yet proposed. We feel quite safe in asserting that it was the most successful of all the modes pursued in the epidemic at Lexington, in 1833, and although the "mammoth" doses of calomel prescribed by Dr. Cooke have been made the subject of much criticism, we are able to testify to many remarkable cases of recovery under that practice. It is by no means our intention to recommend the use of calomel on so large a scale. We believe the quantity given was larger than was necessary; but we wish to be distinctly understood as urging the calomel practice in cholera; and while on the subject it may not be amiss to remark, that the "mammoth" doses have found advocates in other respectable quarters in the profession, and that there is not wanting strong testimony to their value. Pereira* says, "I have now before me reports of eighteen cases of spasmodic cholera, admitted in the year 1832 into the Cholera Hospital at Bethnal Green, in this metropolis, in which enormous quantities of calomel were employed by the house-surgeon, Mr. Charles Bennett, (formerly one of my pupils) with very slight physiological effects. When a patient was brought into the hospital, two drachms of calomel were immediately given, and afterwards one drachm every one or two hours, until some effect was produced. In seventeen out of eighteen cases in which this plan was tried, the vomiting

* Mat. Med. (Am. Ed.) vol. I, p. 615.

and purging diminished, and the patients recovered. Several of them took from twenty to thirty drachms without the subsequent ptyalism being at all excessive. In the unsuccessful case which I have alluded to, fifty-three drachms of calomel were administered within forty-two hours, without the least sensible effect."

On the 12th of December cases of cholera were admitted into the Charity Hospital, at New Orleans, the ship Swanton having arrived from Havre with cases of the disease the day before. The introduction of cholera into New Orleans is ascribed to that vessel, although it did not exist at the port from which she sailed, but broke out, as it did on the New York, during her passage. In the week ending the 16th of December, seventeen deaths were reported from cholera of all kinds, only a few cases being styled *Asiatic.* On the 17th, the Board of Health announced that cases of cholera had appeared in the city, but that they were generally the result of irregularities on the part of the subjects. The weather at this time was damp and hot, the thermometer rising often as high as 80°. Furniture in the houses moulded, and the walls were wet with the hygrometric moisture. Citizens of Louisville, who left New Orleans about that period, describe the condition of the atmosphere as having been intolerably sultry and oppressive. On the 18th of December ten cases were admitted into the Charity Hospital. We notice that on the 16th the interments are reported as, from "Cholera Asiatic, 3; cholera, 4; cholera morbus, 10; dysentery, 6; diarrhea, 2." From the 16th to the 21st, the number of interments from cholera was 87, and on that day, the Board of Health acknowledged that the disease prevalent in the city was *epidemic* cholera.

The announcement of the Board increased the alarm which before had become very great. On the first rumor of the irruption of the disease a panic is reported to have seized upon the students in the medical school, which was naturally soon communicated to other strangers in the city, and the consequence was that the boats coming up the river were crowded with refugees from the epidemic. But at the same time great numbers of strangers were pouring into the city from all the ships arriving from Europe. In one day, and when the mortality of the pestilence was at its height, more than a thousand German emigrants were landed. It is not wonderful that in such weather, and with such an increase of the fit subjects for the epidemic, its mortality should have rapidly increased.

On the 22d of December, the day after the existence of the epidemic was recognized by the Board of Health, the interments were 46, and for the successive days of that month, 71, 84, 77, 54, 61, 92, 84, 77, and 71. The greatest mortality was on the 28th, when the interments amounted to 92, and nearly equalled those from yellow fever in that place when it has been at its worst. On the 30th the weather cleared up with a northwest wind, and the last day of the year the sun shone out, and the air was cool and bracing. The interments on the 1st of January were 67, and the Board of Health announced that the epidemic was abating; but the day following the number mounted up again to 84. On the 3d the interments were 67, on the 4th, 39, and on the 5th, 44. The Board declared that in private practice the disease had been found entirely manageable, and that the fatal cases were furnished by persons of irregular habits, in whom the first symptoms were met by no medical treatment.

The whole number of deaths from cholera in New Orleans, from the 12th of December to the 5th of January, twenty-five days, was eleven hundred and fifteen, an average of more than forty-four a day. Of this number, five hundred and three, or nearly one-half, occurred at the Charity Hospital, indicating pretty clearly the class of population to which the disease owed its chief mortality. As bearing upon this point, we may refer to a letter from a lady connected with the church of a well known and popular minister in New Orleans, stating that not a single death from cholera had taken place in the large congregation of which she was a member. The places of interment indicate the same thing. On the 27th of December the burials in Potters field were 21, and in St. Patrick's 11; while in the Protestant cemetery they were but 3.

An increase in the fatality of the disease, now no longer regarded by the Board of Health as epidemic, is reported to have followed the celebration of the 8th of January. The number of interments from cholera for the week ending the 12th, is said to have been one hundred and fifty-five, or 22 a day, just half the mortality that attended it when it was pronounced epidemic.

It is worthy of remark, that cholera has not swallowed up all other diseases in New Orleans, nor compelled them to "wear its livery," as has been so often remarked of epidemics; but while it has increased the general mortality, other diseases have continued to prevail.—Thus, while the deaths from cholera in a week were 155, from other diseases the deaths in the same period were 135.

The mitigation in the severity of the epidemic so soon consequent upon the change of weather, is instructive. Up to the close of December the heat had been almost tropical, and the air at the same time was surcharged with moisture. It is well understood that such a state of the atmosphere is peculiarly favorable to bowel complaints, and that it has been in weather such as this that cholera has manifested its greatest malignity.

We have nothing to report in reference to the treatment of cholera in New Orleans, and must take another opportunity to communicate the professional experience of that city on this point.

The boats leaving New Orleans after the alarm of cholera began to spread, were crowded with emigrants and persons flying from the disease, many of whom perished on the passage, and a number died after reaching Memphis, Nashville, St. Louis, Louisville, Cincinnati, and even Pittsburgh. One or two are known to have been seized with the disease, and to have died, on the National road beyond Wheeling. The Peytona, bound for Louisville, buried during her passage fourteen victims of cholera, chiefly from among her deck passengers. Dr. Strother speaks of fourteen cases as having occurred in the boat on which he came up from New Orleans, half of which were fatal. But while the mortality was such among the indigent, the irregular, and the neglected, scarcely a cabin passenger died on any of the boats.

On the 24th of December the Savannah arrived at Louisville from New Orleans with three cholera patients, four of her passengers having died with the disease on her way up. In all, from that time to the 18th of January, seventeen cases have been admitted into the Louisville Marine Hospital, of which nine have recovered, six have died, and two are still under treatment. All the patients except two were in a state of collapse when they entered the hospital. None presented the true "rice water" discharges, but the matter evacuated was a yellow fluid, having the appearance of being more or less tinged with bile. One case originated in the wards of the hospital, the subject being a convalescent from intermittent fever. None of the nurses took the disease, nor did it spread among the other patients.

The most prominent symptoms were the following, as reported to us by Mr. Metcalf, assistant to the House Physician:

"The first symptom was thirst, which generally preceded the diarrhea for some hours, and in some cases for days, observing no definite time. This was followed by diarrhea, the discharges generally liquid and of a pale white color, and in most of the cases the first four or five evacuations were attended with tenesmus. In one case the patient had three evacuations of blood and mucus like those in dysentery, being at the time in a state of advanced collapse. In all the cases, I observed a proportional increase in the thirst and diarrhea; when the diarrhea became profuse, the thirst became insatiable, attended with a burning sensation in the stomach. The countenance assumed a cadaverous expression in a few hours after the commencement of profuse diarrhea. The eyes were sunken, congested, and, in some instances thrown up under the lids, concealing almost the whole of the cornea. When this was the case there was invariably some derangement of the mind, and in one case there was delirium and coma. The post-mortem appearances in this case indicated extensive inflammation of the brain. After a longer or shorter period, varying according to the violence of the attack, vomiting commenced. The thirst became insatiable; the vomiting was attended by considerable nausea, which was relieved for a few minutes by throwing up the contents of the stomach, during which time the constant cry of the patient was for water. In a few hours he experienced cramps in the lower extremities, especially in the gastrocnemii muscles, gradually extending to the hands and arms, and then to the muscles of the abdomen and chest. The pain produced by the cramps in violent cases was excruciating, while in others it was very slight, merely flexing the toes and fingers.

The cramps were always either much exaggerated or excited by any movement or change of place made by the patient. The pulse became accelerated, feeble and empty; the respiration was sometimes oppressed and slow (in one case the respirations were only ten in the minute,) in others it was hurried. The extremities became cold, mottled, and corrugated; the face congested, the lips purple, the tongue, in some cases, slightly coated with a leaden hue, and again was heavily coated with a dirty fur. I have observed this latter condition of the tongue to be a very unfavorable sign. In one patient the tongue assumed a typhoid look; viz: was dry, red, and fissured, but this appearance I have only observed in two very malignant cases, both of which proved fatal. The patients generally remained rational to the last, but in every case, without an exception, there was more or less obtuseness of intellect. When questioned, the patients answered slowly, as if they did not exactly comprehend the question.

"In the case which occurred in the hospital, when on the verge of collapse his surface became bedewed with a cold and clammy perspiration.

"In violent and malignant cases, where the vital powers seemed to be overwhelmed by the disease, there was a total suppression of the urinary secretion. In one case this secretion was suppressed for six days previous to death. In others, where there was not a total suppression of urine, and where recovery took place, micturition always gave some pain for several days.

"The stage of collapse set in, generally, about the third day after the attack.

"Diarrhea generally ceased two or three days previviously to death, and was not difficult to check in the stage of collapse, but no relief followed its suspension, but, on the contrary, the patients frequently experienced so much oppression, that I was obliged to reëstablish the evacuations by stimulating enemas."

The following cases, the notes of which were taken by Mr. Metcalf, will illustrate the character of the disease, as well as the treatment pursued at our hospital.

Case I. J. Varing, a native of Ireland, temperate and muscular, was admitted on the 1st of January. He was just from New Orleans, and had come up as a deck-hand on a boat which lost several of its passengers with cholera. Two days previous to his admission into the hospital he was seized with diarrhea, the discharges being copious, light colored, and watery. Vomiting followed in five or six hours, and cramps shortly afterwards came on. When he came to the hospital he was in a collapsed condition — pulseless at the wrist, of bluish color, shrivelled extremities, with slow and laborious respiration. His thirst was insatiable, but his stomach rejected everything; urine had been suppressed for thirty-six hours. Intellectual faculties obtuse, which was a symptom in all the cases treated in the hospital.

He was treated by all the stimulating agents—hot applications, the hot vapor-bath, brandy, capsicum, etc. An enema of acetate of lead and laudanum arrested the diarrhea, and something like reaction came on, but after a brief space the vital powers gave way again, and on the third day death took place. Calomel was given a few hours before he died.

Case II. The subject of this case was also a native of Ireland, of temperate habits, large and muscular. He was a deck-hand on the Yorktown which arrived on the 5th of January, and was admitted the day following. Eight or ten cases of cholera had occurred on board the boat during her passage. This patient was well when he landed in Louisville. The night after, he was taken with profuse diarrhea, which compelled him to leave his bed every ten or fifteen minutes; cramps soon succeeded, and when he was brought to the hospital his extremities were cold and shrivelled, countenance blue, pupils dilated, eyes congested and sunken, pulse barely perceptible at the wrist, respiration slow and distressed, thirst insatiable, tongue coated with a yellow fur.

He was placed in Jennings' vapor-bath; ice was given to allay his thirst; brandy toddy; and capsicum and camphor, grs. v., with opium, gr. ij., every hour. He continued to sink, and died three hours after he was brought to the hospital.

CASE III. Joseph Kleim, a native of Germany, æt. 33 years, was seized with profuse diarrhea on the 4th of January, having been a patient in the hospital since the 19th of the preceding month. He left New Orleans, where he had an attack of intermittent fever, in July. The fever returned in September, and in December he became an inmate of the hospital. Acetate of lead and opium were prescribed for two days, during which the diarrhea persisted, though not with its original profuseness. He had also vomiting and cramps; pulse 110, feeble and empty; thirst insatiable; tongue coated with a white fur; eyes somewhat sunken and heavy.

At 10 o'clock on the evening of the second day he took calomel, grs. v., Dover's powder grs. viii., and capsicum grs. x., and the dose was repeated every hour and a half. The vapor-bath was reapplied, under which his pulse rose, and he became comfortable. Under the use of calomel, quinine, capsicum, and Dover's powder, he recovered.

This patient, it will be remarked, had been nearly a month in the hospital and contracted the disease there; but many such cases, as we shall have occasion presently to state more at length, have occurred in the city.

Nevertheless, it presented all the characteristics of cholera.

Cases IV and V. John and Timothy Odar, both intemperate, had been living at Lake Providence, Louisiana, where they had labored under intermittent fever for three months. About the last of December they took passage on the Gen. Lane, bound for Cincinnati, and reached this city on the 2d of January. Several cases of cholera occurred while they were on board the boat, but the disease had not appeared at Lake Providence when they came away. Timothy was admitted on the 3d laboring under cholera, on the verge of collapse; John came with him to the hospital, and, with the exception of a slight diarrhea, was very well. Two days afterwards he was brought back in the stage of collapse, and died the day following. Timothy recovered.

The treatment is not given in either case; but in respect to treament, we may remark, in general terms, that the plan found most effectual by the House Physician and his assistant was calomel, in combination with quinine. No doubt that the explanation of the success which attended this practice in several of the cases, is to be found in the fact that the subjects of the disease were convalescents from intermittent fever. And this suggests the question, whether in some of the worst attacks of cholera the disease may not be closely allied to congestive, or malignant intermittent, fever. We know that this is the doctrine of some, and whether generally true or not, we have no doubt that there are cases in which the disease is a masked intermittent, and in which quinine is the principal remedy. This thought we consider worthy of being borne in mind by practitioners.

One of the above cases, it will be perceived, originated in the hospital, and several others were attacked after leaving the boats from New Orleans. An individual from North Carolina, by the way of Philadelphia, was seized with cholera on a boat between Cincinnati and this city, and died. In private practice, cases of cholera have been reported, but it is still generally doubted whether they were of the true epidemic character. A little son of Mr. E. Stokes was taken sick on Saturday morning, the 13th of January, with what his parents conceived to be diarrhea, of which he had been the frequent subject, and which, therefore, excited no alarm. But in the afternoon he commenced vomiting, and in the evening it was found that his surface and extremities were growing cold, and his pulse had become imperceptible. The family physician, Dr. Talbot, was called in, and found the condition of the patient to be hopeless; he died about 12 o'clock the same evening. He had no cramps, but the discharges were of the true rice water description, and the symptoms otherwise were those of cholera. At New Orleans, we perceive that the same unwillingness was shown to call the disease Asiatic cholera. Of the ninety-two fatal cases on the 28th of December, four only were reported to be of that type. Several persons have suffered, and some have died, in Louisville, during the last few weeks, with what, if the disease had been epidemic, would have been considered cholera. The cases, however, have been scattering, and there has not at any time been manifested an epidemic tendency in the disease.

The following case has been communicated to us by Dr. D. W. Yandell, and is one of a class of which most of the practitioners of Louisville have seen examples, in the last four or five weeks. At the time that the case of Dr. Y. was under treatment, a great many persons in the city were affected with diarrhea, accompanied in some by vomiting, and cramps, and in all by nausea, and other symptoms of gastric derangement.

"Mrs. S., a married woman, 43 years of age, living near the river, in a small, badly ventilated, and dirty house, after complaining for several days of a looseness of her bowels, was seized during the night of the 6th of January with vomiting, violent cramps, and profuse alvine discharges, which continued with increasing violence till 7 o'clock in the morning, when they abated in some degree, leaving her greatly exhausted, and with strong disposition to sleep. I was sent for immediately after breakfast, but owing to other engagements did not see her till 1 o'clock in the afternoon; two hours before which time the vomiting and cramps, unaccompanied by the diarrhea, had returned and were then occurring at short intervals. She was violently cramped—"twisted into a knot," as her husband expressed it—when I entered the room, throwing up a greenish looking fluid, and soon after had a very copious rice water stool. The surface of her body was of a deep livid hue, and cold, as were also her extremities; the expression of her face anxious; breathing laborious; pulse scarcely perceptible, very frequent, and empty; excessive thirst, the stomach rejecting all liquids as soon as taken.

"Sulphuric ether used internally in teaspoonful doses, and inhaled until the patient was brought fully under its influence, instantly relieved, as by a charm, the cramps, gave softness, volume, and steadiness to the pulse, brought back the blood to the surface, and after its effects had entirely subsided the patient was in every respect comfortable; her pulse only very slightly accelerated, and nearly natural in volume, her skin pleasant, intellectual faculties unclouded, and the disposition to vomit entirely wanting, though she still had repeated calls to stool. Calomel, opium, acetate of lead, and camphor, in liberal doses, followed, after the lapse of sufficient time, by castor oil, secured quiet, put a stop to the diarrhea, brought away two large, black, and very offensive stools, reduced the pulse to its natural standard, and, save considerable exhaustion, left the patient comparatively well.

"The disposition to cramp returned several times during the night, and was as often immediately and entirely overcome by the anæsthetic."

Cases of cholera were translated by the boats from New Orleans to Cincinnati. Professor Lawson, in the Western Lancet for January, remarks—

"Two patients died at the Commercial Hospital, one on the 24th, and the other on the 25th December, the prominent symptoms being vomiting, purging and spasms. The dejections in one case which we saw—or rather the fluids remaining in the intestinal tube after death—were *thin*, but not quite the characteristic *rice water* discharges. Post-mortem examinations, moreover, revealed considerable organic disease of the intestinal canal, sufficient, with exposure and imprudence, to have caused the disease. Still these cases may have been *choleroid*, that is, under some degree of choleraic influence, but not presenting all the symptoms belonging to well-marked cases of that disease.

"Since the death of these two patients, two other cases have occurred in the hospital, presenting symptoms more or less approaching Asiatic cholera, one, indeed, having true rice water evacuations. These cases have not yet terminated. We hear, also, upon good authority, of a case in private practice (from New Orleans) presenting the symptoms of cholera in a still more marked degree.

"Upon the whole it seems probable that patients laboring under cholera influence, received at New Orleans, have reached Cincinnati; but there is certainly no *epidemic* influence manifested here, and it is hardly probable that the disease will extend very rapidly."

The Cincinnati Gazette of Wedneshay, the 10th of January, in noticing the health of Cincinnati, says:

"From all the facts we have been able to gather (and we have taken some pains to ascertain particulars from authentic sources) there have been eighteen deaths from cholera in Cincinnati since Christmas. Twelve of these cases originated upon the river—six were of domestic origin. We reported eight cases of cholera at the hospital on Saturday. There are now but three there, two of which are improving; one not fully developed at last report. On saturday there were two deaths in the hospital; on Sunday three.

"There are now but five cases of cholera in the private practice of physicians within our corporation, known to the Board of Health; these five added to the three in the hospital make eight at present definitely ascertained."

The epidemic appears to have broken out at St. Louis. The newspapers of that city, dated January 18th and 19th, give the following intelligence:

"Five deaths from cholera have appeared in our city during yesterday and the evening previous, and one new case, on Collins street, reported to the health officer. Two of the deaths were in a family on Sixth street, between Locust and St. Charles; one at the corner of Eighth and Wash; one on Wash, between Eighth and Ninth streets; and one at the Sister's Hospital. From two of the families where deaths occurred, several members were sent to the hospital, prostrated with the disease. These, we are informed, are cases of local cholera.

"No additional cases to those previously noticed were reported to the health officer yesterday. Thursday evening two deaths occurred at the Sister's Hospital, being mother and daughter, and making five members of a family who resided on Sixth street, between Locust and St. Charles, that have died of this disease since last Monday noon. We understand that a meeting of the physicians of the city was held on Thursday evening, with a view of consulting in reference to the approach and actual existence of this disease in our city, the result of which we were unable to ascertain. We have no doubt, however, that all actual cases of cholera have been reported to the Board of Health, and that the hundred rumors afloat respecting the increased number of cases in the city are without foundation."

In the week ending Monday the 22d, seventeen deaths are reported to have occurred from cholera in that city.

The present epidemic appears to differ, in one essential feature, from that which invaded our country in 1832. In that, our recollection is, there was little to fear from the subsequent symptoms; if the patient survived the first onset of the disease, his recovery was pretty certain. In this, the typhoid stage which follows the primary symptoms is full of danger. According to the report of Dr. Whiting, most of the patients at Staten Island passed through the first attack, and died from subsequent congestion or effusion of the brain. This has been the case in repeated instances in this city. It is a question worthy of serious consideration, whether the free use of opiates, astringents, and stimulants has not contributed to this result. We have reason to expect that the character of cholera will be modified by the typhoid tendency now existing in the country, and our treatment must have reference to that diathesis. The remark of Dr. Whiting, "that the stimulating plan is not the treatment for *this* cholera" is full of significance.

The probability of the spread of cholera on the opening of spring, is a question of anxious interest, concerning which medical men are often asked their opinions. Referring to the past, our reply would be, that the disease seems to us likely to become epidemic. Everywhere it has pursued very remarkably the same course that it took in its march from East to West, during its former prevalence in Europe and America. We find the same phenomena prevailing now that were its percursors then. In a notice of the diseases of the summer and fall of 1832*, we wrote, that winter, as follows:

* See Transylvania Journal of Medicine, vol. v., p. 501.

"Towards the latter end of June a disposition to bowel complaints manifested itself among the citizens, and for several weeks almost every one complained of a want of appetite, indigestion, and other signs of gastric and intestinal derangement. In some instances vomiting attended, and such other symptoms as required medicine, but generally they yielded to abstinence and rest. Early in September these affections disappeared. They were generally regarded as precursors of cholera, which at the time was raging in New York and elsewhere, and consequently occasioned much uneasiness.

"During the prevalence of these complaints the weather was peculiar. In all the month of June it rained but twice. July was dry until the 20th, when there was a copious fall of rain. But during this whole period, and throughout the month of August, there was very little thunder and lightning. When rain came on, it was not preceded by the usual electrical phenomena. The clouds seemed dead, and though they frequently hung over us for days together, but little rain fell.

"On the 9th of October Dr. Drake announced in the public newspapers that cholera had appeared in Cincinnati. At first it was discredited by the Sanitary Board and the citizens generally, but a few days removed all doubts. The disease spread with terrific rapidity, and in a short time was in every quarter of the city. It was attended with a fatality which had not been equalled in any part of our country, and in but few places in the world. 'It attacked indiscriminately persons of all grades, temperaments and habits of life; and if the rich and temperate suffered less than the poor, the drunken and the exposed, it was not from any immunity against the disease, but from their more carefully watching its first approaches, and from the first stage being milder in its character, and of longer duration.'

"On the 26th of October Dr. Drake writes—'Twenty-seven days have now elapsed since the onset of the pestilence, during which period the whole number of deaths, as far as it has been ascertained, is 351. The greatest number of deaths was from noon on the 19th to noon on the 20th, and amounted to 42, or one out of every six hundred inhabitants actually in the city at the time. Since that day, the mortality has slowly diminished.'

"About the same time the disease appeared in Louisville, but did not spread to the same extent, nor commit such ravages. It was confined to the banks of the Ohio, and the Beargrass, (a creek which empties into the Ohio at this point), not extending to the healthy portions of the city. In a letter to the writer, Dr. Declary remarks: 'On Beargrass, at the point where the Bardstown road crosses it, out of three families consisting of seventeen persons, nine fell victims to its ravages in a few days. At Farmington the seat of Judge Speed, I attended a number of his negroes and several white men under his employ. In the front of his house the ground was low and wet, through which a ditch had been cut. Upon the margin of this ditch a stable had been erected, but latterly this had been converted into a dwelling house for seme of the work hands. Two dissipated laborers had occupied it, both of whom had diarrhœa; the first neglected himself and became the victim of cholera, the other soon followed. Two negroes who waited upon them as well as a young gentleman of the family, were seized, the two former of whom died. Three other deaths followed in the space of thirty-six hours; and a whole family of eighty persons, were more or less under a cholera predisposition, but were cured by timely attention. In Portland, near Louisville, its ravages were great. Along the river, and Beargrass, in one or two points of the Pond settlement, its fatality was truly appalling.'

"The mortality in this city never became great, seven a day being about the average number of deaths reported by the Board of Health, while the disease was at its height.

"The pestilence made its appearance in Frankfort about the 6th of November, and in a population not exceeding 2500, five died in the first forty-eight hours. The severity of the onset induced a belief that it would rage in that place with uncommon violence; but it abated almost at once, and disappeared in a few days without doing much farther mischief.

"Lexington was visited by the disease about the same time. A negro died on the 6th with what was considered by his physicians cholera spasmodica. Other cases occurred on the 8th and 9th, of which two terminated fatally. On the morning of the 9th, William Hutchison, an engineer by trade, of laborious habits, but very poor, was attacked with diarrhœa immediately after breakfast. The morning was cold, and he had been exposed, after being heated over the engine, in thin clothing. After having had a number of very copious rice-water passages, he set out to get medical advice, and walked more than half a mile. He was ordered 20 grs. calomel, with a grain of opium, and to take to his bed. At 2 o'clock I saw him, when he seemed comfortable, having had but a few passages after taking the calomel, his skin being warm and moist, and the slight spasms, with which he had been seized, having left him. About an hour afterwards he had a most copious evacuation of dirty rice-water, after which he was greatly exhausted, and the spasms returned with increased violence. The discharges continued from his bowels, and when I visited him at 5 o'clock I found him in a state of collapse, with pulseless wrists, purple, cold skin, and complaining of blindness. Wine was administered freely, and calomel given in 60 grain doses, repeated at intervals of two hours, till 12 o'clock. His pulse was restored at his wrist, and the warmth of his skin returned. He died next morning at 8 o'clock. One or two men died on this day and the next, when the disease abated. No fatal case occurred after the 11th, and in a few days even mild cases ceased to be presented. In all, but five persons died of the disease—two white men, and three negroes.

"The pestilence has showed itself in most of the towns along the Ohio, from Pittsburg to the mouth, as well as in St. Louis, on the Mississippi, and has travelled down that river to New Orleans, where the reported mortality has been truly appalling. As many as two hundred a day are said to have died for several days, out of a population not exceeding at this time forty thousand souls. The latest accounts state that the scourge is subsiding."

In anticipation of its prevalence, it becomes the duty of physicians to prepare the public in the best way they can for the disease. One of their first obligations, is to allay the fears of people as far as possible. They should be taught that cholera, when taken in time, is a manageable disease, that it is generally preceded by premonitory symptoms, and that safety consists in meeting the complaint in this stage. They should be warned to apply remedies on the first appearance of diarrhea, if the disease should become epidemic, and to seek at once for medical aid. A dose of calomel, taken in brandy; the recumbent posture; hot applications to the feet and general surface; the calomel to be repeated until bilious evacuations appear—these strike us as the proper means to be recommended to people who live remote from physicians. In the course of this article we have indicated the other articles which may be demanded in the progress of the case.

